# Predictive Risk Factors for Low Anterior Resection Syndrome (LARS) in Rectal Cancer—An Observational Cohort Study

**DOI:** 10.3390/jcm14082831

**Published:** 2025-04-19

**Authors:** Sorinel Lunca, Stefan Morarasu, Constantin Osman, Fadi Al Shatarat, Tudor Gramada, Mara Razniceanu, Monica Buzemurga, Emanuel Baltig, Raluca Zaharia, Wee Liam Ong, Gabriel Mihail Dimofte

**Affiliations:** 1 2nd Department of Surgical Oncology, Regional Institute of Oncology (IRO), 700483 Iasi, Romania; sdlunca@yahoo.com (S.L.); fadishatarat1@gmail.com (F.A.S.); gdimofte@gmail.com (G.M.D.); 2 Department of Surgery, Grigore T Popa University of Medicine and Pharmacy, 700115 Iasi, Romaniamara.razniceanu@gmail.com (M.R.); 3 Department of Radiology, Regional Institute of Oncology (IRO), 700483 Iasi, Romania; 4 Department of Radiotherapy, Regional Institute of Oncology (IRO), 700483 Iasi, Romaniaebaltig@yahoo.com (E.B.)

**Keywords:** low anterior resection syndrome, rectal cancer, rectal surgery, radiotherapy, functional outcomes, risk factors, quality of life

## Abstract

**Background/Objectives**: The improved long-term survival of rectal cancer patients has led to a major increase in the prevalence of functional complications. Understanding which patients are prone to develop major LARS is important for their preoperative counselling and follow-up. Herein, we aimed to assess the risk factors for LARS. **Methods**: This is a retrospective cohort study on rectal cancer patients. All patient and tumour variables, management plan, type of neoadjuvant therapy, radiation dose to anal sphincter, and perioperative outcomes were collected from the hospital electronic databases. We quantified LARS and compared the score before and after surgery (mean follow-up of 42.2 ± 32 months). **Results**: A total of 182 patients were included for the final analysis. LARS was present in 43.4% (n = 79) of patients, with 14.8% (n = 27) having minor LARS and 28.5% (n = 52) having major LARS. Age (*p* = 0.03), male gender (*p* < 0.00001), smoking (*p* = 0.04), neoadjuvant radiotherapy (*p* = 0.02), rectal stump length (*p* = 0.008), end-to-end anastomosis (*p* = 0.008), and ileostomy (*p* = 0.002) were found to significantly increase the rate of LARS. A logistic regression model based on the above variables was able to predict major LARS with good predictive value (AUC 0.700). **Conclusions**: LARS is highly common after sphincter-preserving surgery, and it is significantly more common in young, male patients with a history of smoking, having mid-lower rectal cancers with neoadjuvant radiotherapy, and undergoing TME surgery with end-to-end low anastomosis and ileostomy.

## 1. Introduction

Advancements made in the treatment of rectal cancer through better surgery and refined neoadjuvant therapy have led to an unprecedented improvement in disease-free survival and early social reintegration of patients. However, this means patients are more likely to develop long-term functional complications relating to rectal cancer treatment, and so we are witnessing an ever-growing cohort of patients with low anterior resection syndrome (LARS), sexual dysfunction, and urinary dysfunction [1,2,3]. All these complications are more frequent, highly debilitating, and poorly managed outside subspecialized rectal cancer departments. LARS is a common yet often debilitating consequence of sphincter-preserving surgery, characterised by symptoms such as faecal incontinence, urgency, clustering, and incomplete evacuation, usually quantified through the LARS score. LARS significantly impacts patients’ quality of life and is often a permanent issue, sometimes warranting conversion to a stoma [4,5].

It is accepted and expected that LARS is a consequence of total mesorectal excision (TME) surgery, where a low colorectal/coloanal anastomosis is performed; however, the severity of LARS and symptom predominance vary among patients. Moreover, some suggest radiotherapy that has a more profound influence on LARS development compared to surgery [6]. Many risk factors influence LARS severity, including patient-related variables (age, sex, comorbidities), tumour characteristics (location, staging), surgical factors (extent of resection, anastomotic technique), and neoadjuvant therapies (radiotherapy, chemotherapy) [7,8]. However, so far, there is no clear model for predicting LARS severity capable of guiding preoperative patient counselling on their long-term functional expectations. And when considering that reports suggest a better quality of life in patients after Hartman’s TME (permanent stoma) rather than sphincter-preserving surgery (because of LARS) [9], it is reasonable to conduct research on defining risk factors and constructing a model that can predict severe LARS and thus help clinicians and patients better plan surgery.

Herein, we aimed to conduct a comprehensive retrospective analysis on LARS severity and predictive risk factors for LARS development in patients with rectal cancer managed at our institution.

## 2. Materials and Methods

### 2.1. Design and Setting

This is a single-centre, single-department, observational cohort study on rectal cancer patients who were managed at our institution between 2013 and 2024. All patients underwent standard oncological work-up and management based on multidisciplinary meetings. All patients were treated and followed at our institution. LARS was evaluated using the unedited Romanian-validated LARS score, which was quantified through a telephone questionnaire by two investigators who followed a set number of questions. We quantified and compared the score before and after surgery (up to 18 months postoperatively). Besides scoring the LARS in a standard fashion, to deconstruct LARS and see which symptoms predominate in each subgroup of patients, we considered the presence of any symptom as a positive event, while scoring the maximum points for any question was considered as indicating severe symptomatology. The subsequent qualitative variables were analysed to assess the distribution and severity of symptoms among patients with no LARS and those with minor/major LARS. Patient consent was waived by our Institution’s Ethics Committee as the research did not pose any risks to the patients, and the data were anonymised.

### 2.2. Inclusion and Exclusion Criteria

All patients with LAR for rectal cancer who agreed to participate and answered the questionnaire were included in the study. We excluded patients who, at the time of the call, had a diverting stoma, lacked discernment (i.e., unable to answer questions clearly), and patients who had died or did not answer the call.

### 2.3. Data Analysis

Preoperative, intraoperative, and postoperative patient data were extracted from the hospital’s electronic database. An Excel database was created, which included name, sex, diagnosis, preoperative radio-chemotherapy, operation date, intraoperative diagnosis and findings, and patients’ responses to the questionnaire, which included the LARS score and the actual response for each question. Fisher’s test was used to compare events among groups, and a t-test (pooled variance) was used to compare means with their SD. Univariate and multivariate logistic regression were used to assess risk factors. ROC curve analysis was performed to predict each variable’s influence on the outcome. As previously performed, analysis was performed via XLSTAT v.2024.3 software [10,11,12].

## 3. Results

### 3.1. LARS Overview

**Figure 1:**
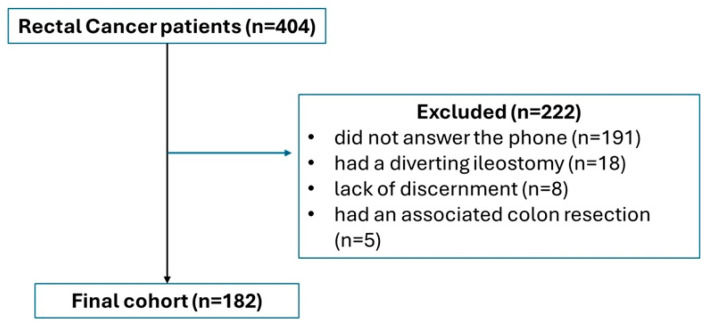
STROBE flowchart.

**Figure 2:**
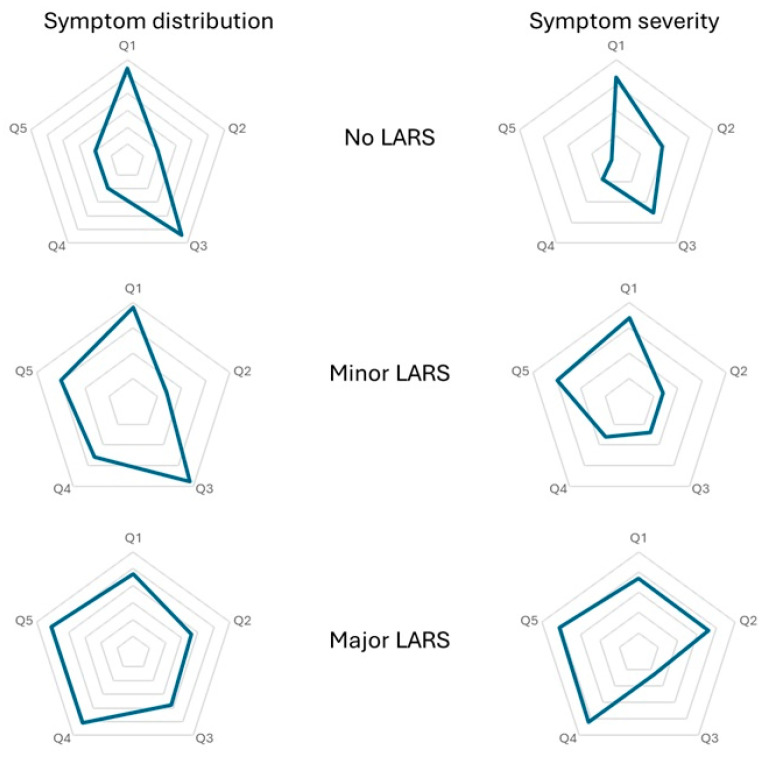
Deconstruction of LARS based on its symptom distribution and severity in each group. The shape of the figure highlights the predominance of each symptom (longer peaks point towards the more common symptoms). Key: Q1: Do you ever have occasions when you cannot control your flatus (wind)?; Q2: Do you ever have any accidental leakage of liquid stool?; Q3: How often do you open your bowels?; Q4: Do you ever have to open your bowels again within one hour of the last bowel opening?; Q5: Do you ever have such a strong urge to open your bowels that you have to rush to the toilet?

After data extraction based on diagnosis, a total of 404 patients were included for triage. The STROBE flowchart (Figure 1) highlights the exclusion criteria, with 182 patients being selected for the final analysis. Overall, LARS had an incidence of 43.4% (n = 79). LARS deconstruction, based on its severity and symptom predominance, is depicted in Figure 2. Despite being classified as no LARS, based on the scoring system, patients in this group still have significant disabilities, receiving scores that indicate medium symptoms on most questions, despite not reaching a total score high enough to be classified as LARS. The most predominant symptoms in patients without LARS were incontinence to flatus (53.3%, with 32% reporting this symptom at least once per week) and frequent bowel movements (52.4%, with 24.2% reporting more than seven bowel movements per day). In patients with LARS, especially major LARS, the most predominant symptoms were clustering (98% with 80.7% reporting clustering at least once per week) and urgency (98% with 78.8% reporting symptoms at least once per week). All symptoms were more frequent in the two LARS subgroups, except frequency of bowel movements (Q3), where patients without LARS had similar symptom severity compared to the other two subgroups (24.2% vs. 25.9% vs. 25%, *p* = 0.982). Only 38.4% (n = 70) of patients received treatment for their symptoms, with 81.1% (n = 57) being prescribed pharmacological treatment with Loperamide, Diosmectite, and stool-bulking agents.

### 3.2. Patients’ Characteristics

For the comparison of the risk factors for LARS development, only the 182 operated patients were analysed, who were split into three groups after completing the LARS questionnaire: control/no LARS (n = 103), minor LARS (n = 27), and major LARS (n = 52). The mean age was 65.8 vs. 66.4 vs. 61.8 years old, with major LARS patients being significantly younger (*p* = 0.039). Also, significantly more males were in the two LARS subgroups (51/49.5% vs. 18/66.5% vs. 30/57.6%, *p* < 0.00001). The BMI, diabetes, or frailty status (measured by the 5-item modified frailty index) were not found to be correlated with worse LARS (*p* = 0.994, *p* = 0.124 and *p* = 0.802); however, smoking was significantly more frequent in patients who developed minor and major LARS (37/35.9% vs. 15/55.5% vs. 28/53.8%, *p* = 0.044). While tumours classified as mid-lower rectum had a higher rate of LARS development compared to superior rectum ones (*p* = 0.013), the distance to the anal verge (AV) measured on preoperative MRI did not correlate with LARS development (92 ± 36.3 mm vs. 86 ± 27.7 mm vs. 87.3 ± 32 mm, *p* = 0.994), the preoperative tumour size (cT3-4, *p* = 0.352), or nodal status (cN2, *p* = 0.115) (Table 1).

### 3.3. Neoadjuvant Therapy

Patients who underwent neoadjuvant long-course radiotherapy (45 Gy and 50.4 Gy boost) had significantly and progressively higher rates of both minor and major LARS (52/50.4% vs. 16/59.2% vs. 38/73%, *p* = 0.026). To assess whether there is a direct correlation between the radiation dose targeted on the anal sphincter complex and LARS development, contouring histograms and dose distributions were evaluated, and the maximum dose on the anal sphincter (Dmax), the mean dose on the anal sphincter (Dmean), the mean dose on half (50%) of the sphincter volume (D50%), and the volume of the sphincter that received 50 Gy (V50) were quantified. None of the dosimetric variables showed a correlation with LARS development (*p* = 0.062, *p* = 0.693, *p* = 0.667, *p* = 0.540) (Table 1).

### 3.4. Operative Factors

Robotic surgery did not show a lower rate of LARS development (*p* = 0.722); however, performing stapled end-to-end anastomosis instead of side-to-end showed a higher rate of both minor and major LARS (49.5% vs. 70.3% vs. 73%, *p* = 0.008). Also, the construction of loop ileostomy was followed by a higher rate of minor/major LARS (54.3% vs. 62.9% vs. 82.6%, *p* = 0.002). The time to the reversal of ileostomy did not correlate with LARS development (*p* = 0.647). While preoperative pelvimetry studies performed using MRI (anteroposterior diameter, sacral curve, and interspinous diameter) were not found to correlate with LARS, the stump length measured on postoperative CT/MRI did influence major LARS development, with no LARS and minor LARS patients having comparable rectal stump lengths (65.2 ± 29.3 mm vs. 65.2 ± 24.6 mm) and major LARS patients having a significantly lower rectal stump length of 53.9 ± 18.7 mm (*p* = 0.008). Besides LARS symptomatology, patients with minor and major LARS also showed a higher rate of urinary dysfunction measured through the International Prostate Symptom Score (IPSS, *p* = 0.018) (Table 1).

### 3.5. Major LARS Prediction

**Figure 3:**
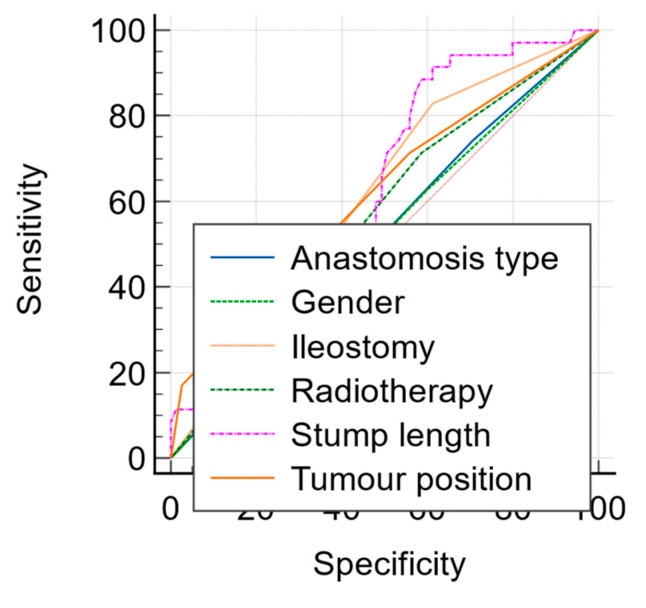
Comparison of ROC curves between six risk factors and their predictive value for major LARS.

**Figure 4:**
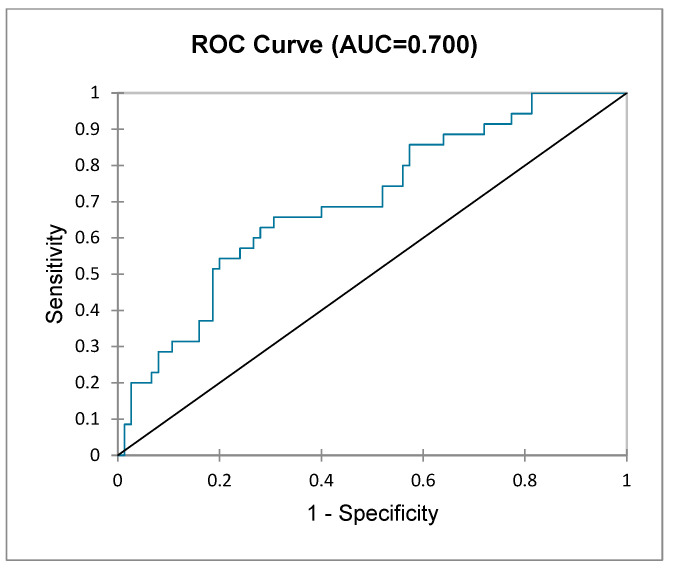
ROC curve showing the predictive value of the constructed model based on each variable’s predictive probability.

Following the univariate analysis, factors that were found to increase the risk of major LARS were extracted, and multivariate logistic regression was performed. The included variables for major LARS prediction were gender, tumour position, neoadjuvant radiotherapy, type of anastomosis (end-to-end vs. side-to-end), ileostomy, and rectal stump length. Figure 3 depicts the sensitivity and specificity of each variable in predicting major LARS, with rectal stump length and tumour position showing the best predictive value (AUC 0.616 vs. 0.608). The predicted probabilities for each variable were included in a new overall prediction model, which increased the predictive value for major LARS to an AUC of 0.700, an acceptable predictive value (Figure 4).

## 4. Discussion

LARS is a highly prevalent long-term functional complication after rectal cancer surgery affecting almost half of patients (43.4%); however, even in the other half of patients still suffer from various bowel dysfunctions despite not scoring enough to be diagnosed with LARS. Frequent bowel movements (more than seven per day) are as common in patients without LARS as in those with LARS, while urgency and clustering are significantly more common in LARS patients, making them prerequisites for LARS diagnosis (Figure 2). Interestingly, patients without LARS report a 53.3% rate of incontinence to flatus vs. 70.3% and 90.3% in patients with minor and major LARS, respectively; this is an unexpectedly high rate (although significantly lower than patients with LARS) of incontinence to flatus in the no LARS group. Even so, incontinence to liquids, which is the most debilitating symptom, is clearly more present in major LARS patients (69.2%) vs. patients without LARS, where the rate is only 18.4%.

When considering risk factors to predict LARS, it seems that younger males with a history of smoking and a mid-lower rectal cancer who undergo neoadjuvant radiotherapy followed by TME surgery with end-to-end anastomosis and ileostomy have the highest risk of developing major LARS. Rectal stump length, measured on postoperative imaging, especially when lower than 30 mm, is also a significant predictor of major LARS. Bowel dysfunction is also associated with a significant rate of urinary dysfunction. The rate of urinary dysfunction measured through the IPSS score is directly proportional to the increase in severity of LARS. While one would expect an increase in LARS in patients with lower vs. upper rectal cancers and with radiotherapy vs. those without, this study brings new insights into constitutional risk factors such as gender, as males seem to have a higher rate of LARS, and age, as younger patients report worse LARS. It is difficult to say whether younger patients really have worse symptoms or LARS has a greater impact on their quality of life because they are more active socially and professionally, so younger patients are more aware of their symptoms. Patients with loop ileostomy showed higher LARS rates than those without. Although relevant, the construction of an ileostomy may simply be a confounding factor related to the tumour position and history of radiotherapy, considering that almost all patients with low cancers and neoadjuvant radiotherapy would have an ileostomy fashioned in our institution. Interestingly, when end-to-end low stapled colorectal anastomoses are performed instead of side-to-end ones, the rate of both minor and major LARS increases significantly. This is an unexpected finding when compared to previous studies, which showed similar functionality between the two techniques, although it would make sense considering that through a side-to-end anastomosis, a larger proximal ampulla is created, which could delay colon emptying.

We would expect that the radiotherapy dose on the sphincter (mean, max, and how much of the sphincter’s volume received the dose) to have an impact on LARS severity, but with our data, this could not be proven. Some studies have shown that measuring pelvic diameters (anteroposterior, sacral curve, interspinous) can predict the difficulty of surgery, operative time, and quality of specimen resection [13,14,15]. Theoretically, in a narrower pelvis, the risk of injuring the lateral pelvic nerves could increase and determine worse functional outcomes; however, this is disproven by our study. None of the pelvimetric variables correlated with the presence or severity of LARS.

The rate of sphincter-preserving surgery has increased exponentially thanks to refined techniques and better multidisciplinary management of low rectal tumours, and this has led to an ever-increasing number of patients suffering from debilitating bowel dysfunction, with many of them reporting poor quality of life. LARS is so common that one might declare it an expected consequence rather than a complication of surgery, hence the reason why various studies are performed to better understand the risk estimation of LARS [16,17] and how we should better prevent and manage it. So far, the only risk predictor of LARS was published by Battersby et al. (2016) [18] in the form of a free online calculator (POLARS) consisting of age, gender, TME/PME, tumour height, stoma, and neoadjuvant radiotherapy. It is easy to use, but in recent studies, it was shown to fail in predicting major LARS, leading a group of authors from Sweden’s Karolinska Institute to conclude that other methods to predict LARS should be developed after testing POLARS’s predictive value on their cohort of rectal cancer patients [19,20]. Even individual variables proposed as risk factors for LARS have been dismissed in recent studies. Parnasa et al. (2024) [16] surprisingly showed no association between LARS and the use of neoadjuvant radiotherapy in their cross-sectional study. On the other hand, a recent second analysis of the ROLARR RCT [21] showed that the ASA score (more than 1), BMI, and use of adjuvant therapy were better predictors than age, gender, or neoadjuvant therapy, which is again, surprising. The ROLARR RCT analysis is also the only study in the literature we found to assess whether robotic surgery has any impact on LARS occurrence, and it showed, like our study, that it does not correlate with improved LARS scores; it is also the only study that attempted to correlate urinary dysfunction (IPSS) to LARS; however, they could not find an association, whereas, herein, moderate/severe urinary dysfunction was significantly more frequent in patients with LARS. The conflicting evidence reiterates the value of this current analysis and, more so, it highlights the need for further multicentric research on large cohorts to establish a strong risk prediction nomogram.

This study has its limitations, considering that it is a retrospective analysis and thus prone to selection, recall, and misclassification biases, although the data were extracted by a higher surgical trainee and verified by two senior surgeons. Questionnaires were performed by a higher surgical trainee capable of quality interpretation of answers. The low number of patients for some subgroup analyses produced outcomes with a low number of observations, frequently less than five, affecting the statistical power of our analysis. To reduce measuring bias, all the radiological variables (e.g., distance to AV, pelvimetry) were measured by an experienced consultant radiologist from our tertiary cancer centre. Also, the contouring histograms and dose distribution on the sphincter volume were performed by a senior trainee in radiation oncology and verified by two consultant radiation oncologists. Unfortunately, many patients (n = 190) were excluded because they did not answer the phone; this is also a selection bias that may have influenced the overall results and reflection of real-world outcomes.

## 5. Conclusions

LARS is a highly common consequence of sphincter-preserving surgery, and its incidence is significantly higher in younger, male patients with a history of smoking and a mid-lower rectal cancer who undergo neoadjuvant radiotherapy followed by TME surgery with end-to-end anastomosis and ileostomy. Together, these risk factors seem to have the highest predictive value in predicting major LARS.

## Figures and Tables

**Table 1:** LARS comparison based on perioperative variables (presumed as potential risk factors).

Variable	No LARS n (%)/Mean (SD)	Minor LARS n (%)/Mean (SD)	Major LARS n (%)/Mean (SD)	*p* Value
**Total**	**103 (100)**	**27 (100)**	**52 (100)**	
**Age**	65.8 (10.2)	66.4 (7.3)	61.8 (10.1)	***p* = 0.039**
**Males**	51 (49.5)	18 (66.6)	30 (57.6)	***p* < 0.00001**
**BMI**	27.4 (4.6)	28.1 (5.2)	28 (4)	*p* = 0.994
**Diabetes**	17 (16.5)	8 (29.6)	6 (11.5)	*p* = 0.124
**Frail**	42 (40.7)	11 (40.7)	24 (46.1)	*p* = 0.802
**Smoking**	37 (35.9)	15 (55.5)	28 (53.8)	***p* = 0.044**
**Mid-lower rectum tumour**	50 (48.5)	14 (51.8)	38 (73)	***p* = 0.013**
**Distance to AV (mm)**	92 (36.3)	86 (27.7)	87.3 (32)	*p* = 0.994
**Staging**				
cT3-4	62 (60.1)	20 (74)	35 (67.3)	*p* = 0.352
cN2	40 (38.8)	16 (59.2)	26 (50)	*p* = 0.115
**Neoadjuvant CHT**	71 (68.9)	22 (81.4)	43 (82.6)	*p* = 0.120
**Neoadjuvant RT**	52 (50.4)	16 (59.2)	38 (73)	***p* = 0.026**
**Radiotherapy dose**				
Dmax (Gy)	48.6 (9.6)	48.3 (5.6)	46.6 (13.2)	*p* = 0.062
Dmean (Gy)	33.1 (13.8)	33.8 (17.2)	35.5 (15.3)	*p* = 0.693
D50% (Gy)	34.3 (17.2)	33.5 (20.4)	34.4 (19)	*p* = 0.667
V50 (%)	29.2 (33)	30(42.9)	31.6 (35.8)	*p* = 0.540
**Robotic surgery**	14 (13.5)	3 (11.1)	9 (17.3)	*p* = 0.722
**Operative time**	212.6 (73.3)	213.2 (84.1)	212.2 (69.5)	*p* = 0.957
**Anastomosis**				
End-to-end	51 (49.5)	19 (70.3)	38 (73)	***p* = 0.008**
Side-to-end	31 (30)	8 (29.6)	14 (26.9)	*p* = 0.917
**LOS**	9.2 (4.7)	10 (7.8)	9.8 (7.1)	*p* = 0.897
**Ileostomy**	56 (54.3)	17 (62.9)	43 (82.6)	***p* = 0.002**
**Time to reversal (days)**	103.1 (89.1)	80.1 (66.3)	97.6 (128.7)	*p* = 0.647
**Pelvimetry**				
Rectal stump length	65.2 (29.3)	65.2 (24.6)	53.9 (18.7)	***p* = 0.008**
Anteroposterior	113.1 (12.8)	111.3 (11.2)	113.6 (11.9)	*p* = 0.595
Sacral curve	32.5 (9.1)	33.2 (10)	29.8 (8.5)	*p* = 0.082
Interspinous	100.2 (12.1)	97.9 (10.6)	99.5 (11.3)	*p* = 0.210
**Follow-up (months)**	43.3 (31.8)	46.6 (37.1)	33.9 (30.4)	*p* = 0.079
**Moderate/severe IPSS**	37 (35.9)	15 (55.5)	30 (57.6)	***p* = 0.018**

Key: LARS, low anterior resection syndrome; BMI, body mass index; CHT, chemotherapy; RT, radiotherapy; IPSS, International Prostate Symptom Score; Dmax, maximum dose on sphincter; Dmean, mean dose on sphincter; D50%, dose on 50% of the sphincters volume; V50, volume of sphincter that received 50 Gy. Significant variables and results are in bold.

## Data Availability

The dataset is available upon request from the authors.

## Abbreviations

The following abbreviations are used in this manuscript:

LARS	Low Anterior Resection Syndrome
AUC	Area Under the Curve
TME	Total Mesorectal Excision
SD	Standard Deviation
IPSS	International Prostate Symptom Score
BMI	Body Mass Index
AV	Anal Verge
CHT	Chemotherapy
RT	Radiotherapy
Dmax	Maximum Dose on Sphincter
Dmean	Mean Dose on Sphincter
D50%	Dose on 50% of the Sphincter’s Volume
V50	Volume of Sphincter that Received 50 Gy
LOS	Length of Stay
ICU	Intensive Care Unit
